# Favorable outcomes of de novo advanced phases of pediatric chronic myeloid leukemia in the tyrosine kinase inhibitor era

**DOI:** 10.1007/s12185-025-03953-x

**Published:** 2025-03-15

**Authors:** Toshihide Yoshikawa, Hisashi Ishida, Akihiro Watanabe, Yuki Yuza, Haruko Shima, Masaki Ito, Yukari Sakurai, Dai Keino, Takuya Ichimura, Keisuke Kato, Yuko Osugi, Shosuke Sunami, Kunihiro Shinoda, Toshihiko Imamura, Katsuyoshi Koh, Yuri Okimoto, Chikako Tono, Hiroyuki Shimada, Akihiko Tanizawa

**Affiliations:** 1 Department of Pediatrics, Faculty of Medical Sciences, University of Fukui, Fukui, Japan; 2 Department of Pediatrics, Okayama University Hospital, Okayama, Japan; 3 Department of Pediatrics, Niigata Cancer Center Hospital, Niigata, Japan; 4 Department of Hematology/Oncology, Tokyo Metropolitan Children’S Medical Center, Tokyo, Japan; 5 Department of Pediatrics, Keio University School of Medicine, Tokyo, Japan; 6 Department of Pediatrics, Soma General Hospital, Fukushima, Japan; 7 Department of Pediatrics, Asahikawa Medical University, Asahikawa, Japan; 8 Division of Hematology/Oncology, Kanagawa Children’s Medical Center, Yokohama, Japan; 9 Department of Pediatrics, Yamaguchi University Graduate School of Medicine, Ube, Japan; 10 Division of Pediatric Hematology and Oncology, Ibaraki Children’S Hospital, Mito, Japan; 11 Department of Pediatric Hematology/Oncology, Osaka City General Hospital, Osaka, Japan; 12 Department of Pediatrics, Japanese Red Cross Narita Hospital, Narita, Japan; 13 Department of Pediatrics, Gifu Municipal Hospital, Gifu, Japan; 14 Department of Pediatrics, Graduate School of Medical Science, Kyoto Prefectural University of Medicine, Kyoto, Japan; 15 Department of Hematology/Oncology, Saitama Children’s Medical Center, Saitama, Japan; 16 Department of Hematology/Oncology, Chiba Children’S Hospital, Chiba, Japan; 17 Department of Nursing Science, Hirosaki University Graduate School of Health Sciences, Hirosaki, Japan; 18 Department of Pediatrics, Sugita Genpaku Memorial Obama Municipal Hospital, 2-2 Ohtecho, Obama City, Fukui 917-8567 Japan

**Keywords:** Pediatric hematology/oncology, CML, Advanced phase, Stem cell transplantation, Tyrosine kinase inhibitor

## Abstract

Chronic myeloid leukemia (CML) is a rare disease during childhood, and accelerated phase (AP) and blast phase (BP) CML, also called advanced phases, are even rarer. We retrospectively collected and analyzed clinical data of children younger than 20 years with de novo advanced-phase CML between 1996 and 2017 in Japan. Median follow-up time was 8.9 years for AP-CML (n = 15) and 3.7 years for BP-CML (n = 32). The 5-year overall survival (OS) was 93.3% for AP-CML, and 100.0% for patients who received tyrosine kinase inhibitors (TKIs) in first-line therapy (n = 10). Four of the ten patients who received TKIs in first-line therapy remained in molecular remission without transplantation (median follow-up 5.5 years). The 5-year OS of patients with BP-CML was 79.0%, and most patients received chemotherapy before transplantation, with regimen selection based on blast immunophenotype. Furthermore, among patients who received transplantation after TKI therapy, the 5-year OS was 100.0% for AP and 84.8% for BP. In conclusion, our study confirmed excellent outcomes in children with de novo advanced-phase CML, especially in the TKI-era.

## Introduction

Chronic myeloid leukemia (CML) is a rare disease that accounts for 2–3% of leukemia cases in children [1]. CML is characterized by the translocation t(9; 22) (q34; q11.2), which creates the Philadelphia chromosome, leading to the production of the oncogenic *BCR::ABL1* transcript. Most patients with CML are diagnosed in the chronic phase (CP), whereas few patients are diagnosed in the accelerated phase (AP) or blast phase (BP), called de novo AP and de novo BP, respectively. These are called advanced-phase CML, and the criteria for the advanced phase include blast count in the bone marrow or peripheral blood, increased basophil level, splenomegaly, abnormal cytogenetics, or extramedullary disease [2]. The introduction of the tyrosine kinase inhibitor (TKI), imatinib, and the second-generation TKIs (2G-TKIs), dasatinib and nilotinib, has drastically changed the treatment strategy for CML. In Japan, imatinib became available for the treatment of CML in 2001, and 2G-TKIs were approved in 2009. Children with CP-CML are typically treated with TKI monotherapy, whereas those with advanced-phase CML are treated with a combination of chemotherapy and TKIs. However, the optimal chemotherapy regimens to be used in combination with TKIs in children with advanced-phase CML are not well understood.

CML is a rare disease in childhood, and de novo AP-CML or BP-CML is even rarer. Therefore, the experience in children is extremely limited [3–5]. Recently, Millot et al. reported the patient information from a large international cohort, I-CML-Ped [6], with 19 and 17 patients with de novo AP and BP, respectively. They reported excellent outcomes in both groups; the 5-year overall survival (OS) was 94% (95% confidence interval [CI] 65–99%) and 74% (95% CI 44–89%) for de novo AP-CML and BP-CML, respectively. Furthermore, they reported that 13 of 19 patients with AP-CML and 6 of 17 patients with BP-CML did not undergo hematopoietic stem cell transplantation (HSCT), and only one patient with AP-CML and one patient with BP-CML died. This is a remarkable result, as HSCT was generally recommended for all patients with de novo AP- or BP-CML previously [7]. However, because the sample size was small, further validation with other cohort studies is needed to support the strategy of avoiding HSCT in children with de novo advanced-phase CML.

This study aimed to report the characteristics, treatment details, and survival outcomes of pediatric patients with de novo AP-CML and BP-CML with or without TKI administration in Japan.

## Methods

The current study comprised two cohorts with the different collection periods, both of which were retrospectively collected by sending case report forms to the treating institutes of Japanese Pediatric Leukemia/Lymphoma Study Group (JPLSG), which were merged with other pediatric solid cancer groups in Japan and were reorganized as the Japan Children’s Cancer Group (JCCG) in 2017. The first retrospective survey (hereafter referred to as *cohort A*) included children aged < 20 years in all phases of CML and diagnosed between 1996 and 2011. Although this survey has been reported, the previous study was mainly focused on CP-CML[8]. The second retrospective survey (*cohort B*) included only those aged < 20 years with de novo advanced-phase CML diagnosed between 2012 and 2017. Both surveys were conducted by the CML Committee of JPLSG/JCCG. The first study was approved by the Ethics Committee of Keio University School of Medicine, the second study by the Ethics Committee of Niigata Cancer Center Hospital, and the follow-up study of cohort A by the Ethics Committee of the University of Fukui. Informed consent was obtained in the form of an opt-out option on the website. Those who rejected were excluded. The diagnosis of CML was confirmed by the detection of t(9;22)(q34;q11.2) by chromosome G banding analysis or fluorescence in situ hybridization, and the expression of *BCR::ABL1* chimera messenger RNA was determined by reverse transcription polymerase chain reaction or transcription-mediated amplification and hybridization protection assay (TMA-HPA) [9, 10]. AP-CML and BP-CML were diagnosed based on the Japanese guidelines, which were basically identical to those from the contemporary European LeukemiaNet recommendation or guideline [11, 12]. The research protocol stipulated that pathological confirmation through biopsy was mandatory in principle for CML cases with extramedullary lesions only. The immunophenotype, whether lymphoid or myeloid, was determined using flow cytometry in patients with BP-CML. Hematologic, cytogenetic, and molecular responses were defined according to the ELN definition [12]. As for the *BCR::ABL1* transcript level, reduction to 100 copies/μg RNA with TMA-HPA was regarded as approximately equal to a three-log reduction in the international scale (IS) % *BCR::ABL1/ABL1* [10]. Therefore, in the TMA-HPA, a *BCR::ABL1* transcript level of less than 100 copies /μg RNA was considered a major molecular response (MMR), whereas an undetectable level was considered a complete molecular response (CMR). MMR, molecular response (MR) 4.0, and MR4.5 were defined as IS% *BCR::ABL1/ABL1* ratios < 0.1%, < 0.01%, and < 0.0032%, respectively[12, 13]. MR4.0 was considered equivalent to CMR. The probabilities of OS were calculated using Kaplan–Meier estimators, and a univariate analysis was performed using the log-rank test. All statistical analyses were performed using EZR (Saitama Medical Center, Jichi Medical University, Saitama, Japan) [14], which is a graphical user interface for R (R Foundation for Statistical Computing, Vienna, Austria). More precisely, it is a modified version of the R commander designed to add statistical functions frequently used in biostatistics.

## Results

### Background characteristics of the included patients

In cohort A, which included children in all disease phases of CML between 1996 and 2011 in Japan, 228 patients with CP-CML (89.1%); 11, AP-CML (4.3%); and, 17, BP-CML (6.6%) were included, but only patients with AP-CML and BP-CML were addressed in this study. The number of patients was slightly different from that in the original report [8], as the disease phase of one patient previously diagnosed with BP-CML was re-evaluated and changed to AP-CML. In cohort B, four patients with AP-CML and 15 patients with BP-CML were included. Therefore, 15 and 32 patients with AP-CML and BP-CML, respectively, were analyzed in the current study. The patient characteristics are shown in Table 1. The median age at diagnosis was 8y11m (3y9m–19y0m) and 9y7m (1y1m–16y1m) in the patients with AP-CML and BP-CML, respectively. The male-to-female ratio was 2:1 and 1:1 in the patients with AP-CML and BP-CML, respectively. Among the patients with available data on spleen size, splenomegaly was observed in 7/11 (63.6%) and 15/17 (88.2%) patients with AP-CML and BP-CML, respectively. Among those with a palpable spleen, the median size of the spleen below the costal margin was 10 cm (4–20 cm) and 8.5 cm (1–36 cm) in patients with AP-CML and BP-CML, respectively.

**Table 1:** Clinical characteristics of children with AP-CML and BP-CML at diagnosis

	AP (n = 15)	BP (n = 32)
Age: median (range)	8y11m (3y9m–19y0m)	9y7m (1y1m–16y1m)
Sex		
Male	10 (66.6%)	16 (50.0%)
Female	5 (33.3%)	16 (50.0%)
Splenomegaly (%)	7/11 (63.6%)	15/17 (88.2%)
Median (cm) below the costal margin^a^	10 (range 4–20)	8.5 (range 1–36)
Leukocytes (× 10^9^/L)	157.2 (range 8.5–507.6)	184.4 (range 0.9–951.0)
Hemoglobin (g/dL)	9.1 (range 5.6–13.0)	8.4 (range 3.7–13.4)
Platelets (× 10^9^/L)	362 (range 52–1060)	218 (range 16–1638)
Blasts in blood (%)	5.5 (range 0.0–21.0)	24.0 (range 0.0–92.1)
Blasts in bone marrow (%)	3.2 (range 0.0–28.8)	44.6 (range 0.0–99.0)
Immunophenotype		
Lymphoid	–	16 (50.0%)
Myeloid	–	10 (31.3%)
Mixed	–	1 (3.1%)
Unknown	–	5 (15.6%)
Extramedullary involvement	–	11 (34.4%)

*AP* accelerated phase, *BP* blast phase, *CML* chronic myeloid leukemia

^a^Available data: n = 11 for AP and n = 16 for BP

Among the 27 patients with BP-CML with available immunophenotypic data, 16 (59.3%) showed a lymphoid phenotype; 10 (37.0%), a myeloid; and 1 (3.7%), a mixed phenotype. Eleven patients with BP-CML (34.4%) had extramedullary involvement, and data on 11 sites in nine patients were available: skin/subcutaneous: 6; bone: 2; and testis, iliopsoas muscle, or ocular fundus: 1 each. Among the 11 patients with BP-CML with extramedullary involvement, 10 patients (90.1%) did not meet the criteria of BP-CML with their bone marrow findings; these patients met the criteria of BP-CML based solely on the presence of extramedullary involvement.

### Treatment details and outcomes of patients with AP-CML

**Fig. 1:**
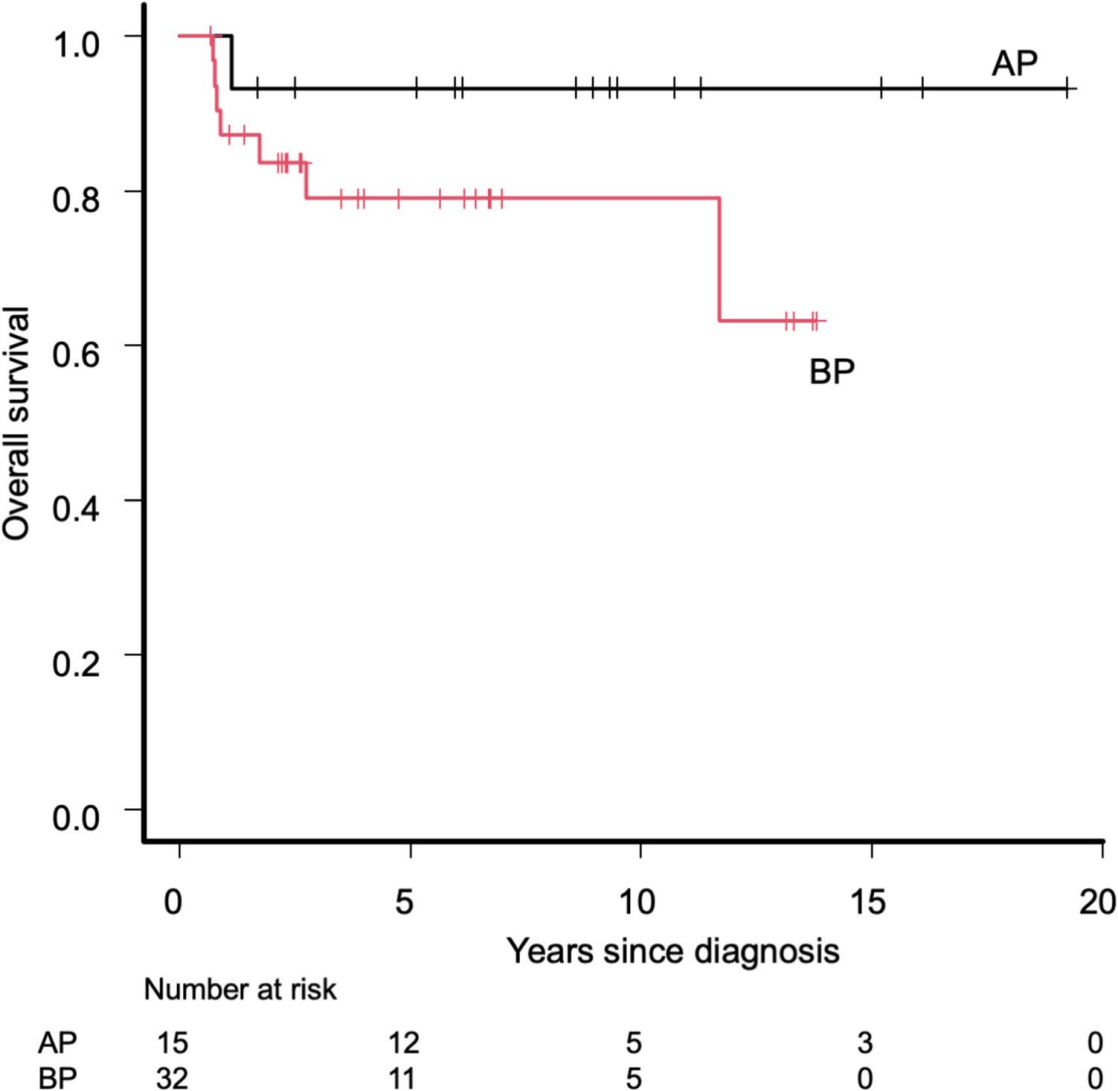
Overall survival of included patients according to disease phase. *AP* accelerated phase, *BP* blast phase

**Fig. 2:**
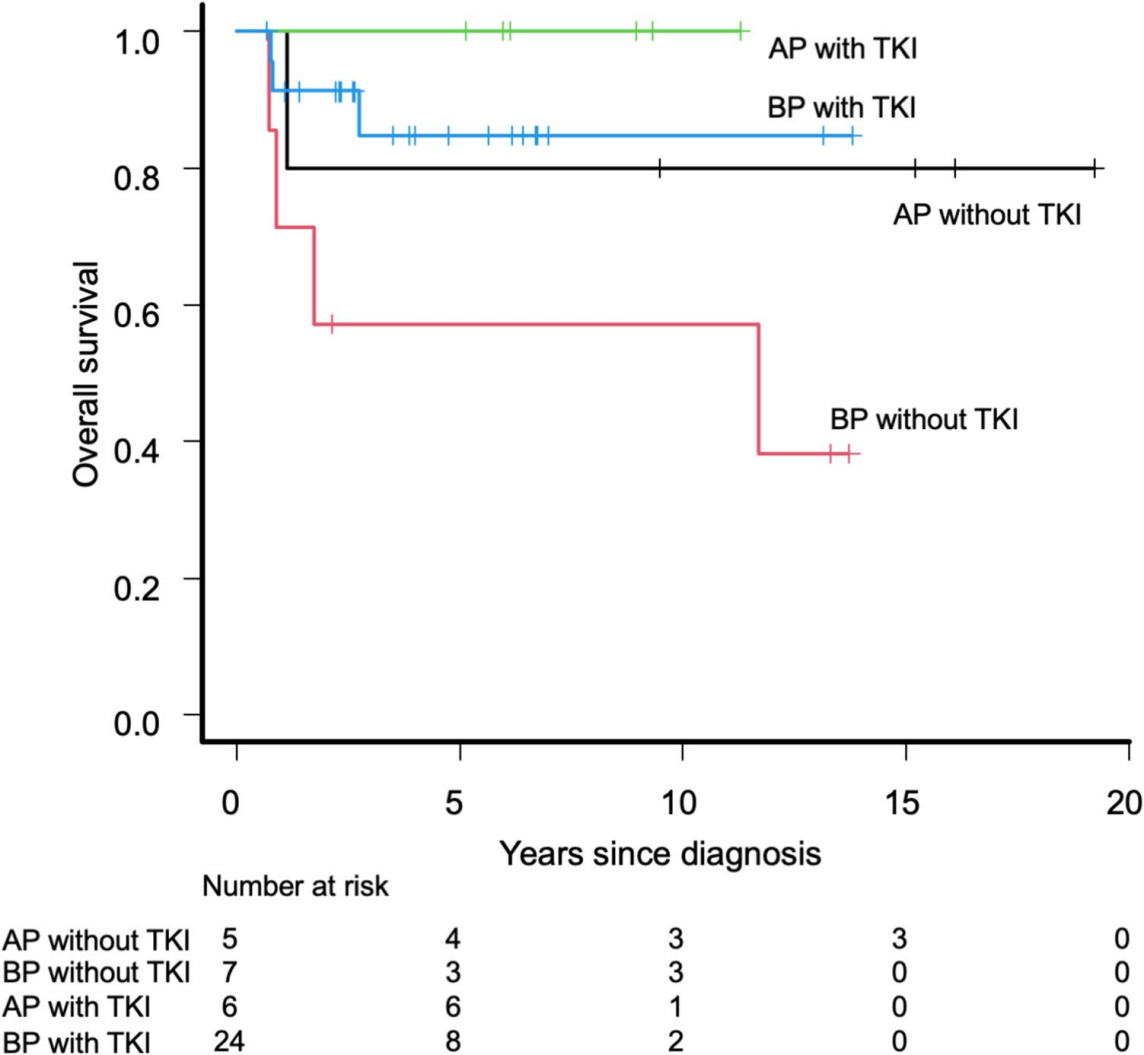
Overall survival of included patients according to disease phase and pre-HSCT administration. *AP* accelerated phase, *BP* blast phase, *HSCT* hematopoietic stem cell transplantation, *TKI* tyrosine kinase inhibitor

Among the 15 patients with de novo AP-CML, 10 patients received TKIs as first-line therapy, and five patients did not receive TKIs because they were diagnosed before TKIs became available (Table 2). With a median follow-up period of 8.9 years (range 1.1–19.2 years), the probability of 5-year OS of the patients with de novo AP-CML was 93.3% (95% CI 61.3–99.0%) (Fig. 1). Among those who received HSCT, the 5-year OS was 100.0% (95% CI, NA) for patients with pre-HSCT TKI administration and 80.0% (95% CI 20.4–96.9%) for patients without pre-HSCT TKI administration (Fig. 2).

**Table 2:** Patient characteristics and outcomes for children with de novo AP

Pt No.	Age/sex	Pre-HSCT TKIs	Pre-HSCT treatment	Pre-HSCT state	Conditioning	Graft	Donor	Post-HSCT TKIs	Current treatment	Final CML state	Outcome
AP-1	9/M	No	HU	CHR	MAC	BMT	MSD	ima^a^	TKI–	MR4.5	Alive
AP-2	3/M	No	HU, IFNα	CHR	MAC	BMT	MSD	No	TKI–	CMR	Alive
AP-3	7/M	No	HU, IFNα	CHR	MAC	BMT	MMRD	No	(NRM; TMA after 2nd HSCT)	CMR	Dead
AP-4	11/M	No	AML-type chemo	CHR	MAC	BMT	MUD	No	TKI–	MR4.5	Alive
AP-5	10/M	No	HU, IFNα	PCyR	MAC	BMT	MUD	No	TKI–	CCyR	Alive
AP-6	8/M	Yes	Imatinib	–	–	–	–	–	TKI +	MR4.5	Alive
AP-7	19/F	Yes	Imatinib	–	–	–	–	–	TKI +	MR4.5	Alive
AP-8	8/F	Yes	Imatinib	PHR	MAC	BMT	MSD	ima^a^	2nd HSCT, TKI–	MR4.5	Alive
AP-9	14/F	Yes	Imatinib + HU, IFNα	CCyR	RIC	BMT	MUD	ima^a^	TKI +	CCyR	Alive
AP-10	5/M	Yes	ALL-type chemo + Imatinib	MMR	MAC	BMT	MUD	No	TKI–	MR4.5	Alive
AP-11	12/M	Yes	Imatinib	MMR	RIC	BMT	MSD	No	TKI–	CMR	Alive
AP-12	8/M	Yes	Imatinib, Dasatinib	CHR	RIC	BMT	MMRD	dasa → nilo → dasa^a^	TKI–	MR4.5	Alive
AP-13	6/F	Yes	ALL-type chemo + Imatinib/Dasatinib	CCyR	MAC	BMT	MSD	No	2nd HSCT, TKI +	MMR	Alive
AP-14	12/M	Yes	Dasatinib	–	–	–	–	–	TKI +	MR4.5	Alive
AP-15	4/F	Yes	Dasatinib	–	–	–	–	–	TKI +	MR4.0	Alive

*AP* accelerated phase, *Pt* patient, *HSCT* hematopoietic stem cell transplantation, *TKIs* tyrosine kinase inhibitors, *CML* chronic myeloid leukemia, *M* male, *F* female, *HU* hydroxyurea, *IFNα* interferon α, *AML* acute myeloid leukemia, *ALL* acute lymphoblastic leukemia, *CHR* complete hematologic response, *PCyR* partial cytogenetic response, *PHR* partial hematologic response, *CCyR* complete cytogenetic response, *MMR* major molecular response, *MAC* myeloablative conditioning, *RIC* reduced intensity conditioning, *BMT* bone marrow transplantation, *MSD* matched sibling donor, *MMRD* mismatched related donor, *MUD* matched unrelated donor; ima, imatinib; dasa, dasatinib; nilo, nilotinib, *NRM* non-relapse mortality, *TMA* thrombotic microangiopathy, *CMR* complete molecular response

^a^TKIs were initiated after confirming disease recurrence (therapeutic)

Among five patients who did not receive TKIs as the first-line therapy, four patients received hydroxyurea ± interferon-α, and one patient received acute myeloid leukemia (AML)-type chemotherapy, all followed by bone marrow transplantation (BMT) from a human leukocyte antigen (HLA)-matched sibling donor (MSD) (n = 2), an HLA-matched unrelated donor (MUD) (n = 2), or an HLA-mismatched related donor (MMRD) (n = 1). The median time between diagnosis and transplantation was 296 days (111–389 days). The disease status at transplantation was complete hematologic response (CHR) in four patients, whereas it was partial cytogenetic response (PCyR) in one patient. Four of five patients without pre-HSCT TKI administration survived for more than 9 years, although only one of them received post-HSCT TKI after a loss of complete cytogenetic response (CCyR). One patient who received BMT from an MMRD experienced graft rejection, underwent peripheral blood stem cell transplantation (PBSCT) as a stem cell rescue, and died of thrombotic microangiopathy. He was the only patient who died at the last follow-up among those with de novo AP-CML.

Among the 10 patients who received TKIs as first-line therapy, eight patients achieved CHR with first-line TKIs (imatinib, n = 6 and dasatinib, n = 2). Among them, two patients received a combination of acute lymphoblastic leukemia (ALL)-type chemotherapy and TKIs, whereas two patients were treated with imatinib as first-line therapy and required second-line therapy because of progression to lymphoid BP (n = 1; AP-13) or AP (n = 1; AP-12). Notably, four patients (imatinib, n = 2; dasatinib, n = 2) did not undergo HSCT and achieved deep molecular remission without disease progression (MR4.0, n = 1 and MR4.5, n = 3). Six patients underwent BMT from MSD (n = 3), MUD (n = 2), and MMRD (n = 1), at a median of 338 days (range 161–691 days) after the diagnosis of AP-CML. They were transplanted in partial hematologic response (PHR) (n = 1), CHR (n = 1), CCyR (n = 2), and MMR (n = 2). In two patients, TKI administration could be stopped after transplantation. One patient who underwent transplantation during PHR experienced loss of CHR, and received imatinib. She received HLA-haploidentical transplantation from her mother and survived with MR4.5 at the last follow-up.

### Treatment details and outcomes of patients with BP-CML

Among the 32 patients with de novo BP-CML, 25 received TKIs as first-line therapy and seven did not because they were diagnosed before TKIs became available (Table 3) Considering a median follow-up period of 3.7 years (range 0.7–13.8 years), the probability of 5-year OS of the patients with de novo BP-CML was 79.0% (95% CI 58.6–90.1%) (Fig. 1). Among those who received HSCT, the 5-year OS was 84.8% (95% CI 58.9–95.0%) in patients with pre-HSCT TKI administration and 57.1% (95% CI 17.2–83.7%) in patients without pre-HSCT TKI administration (Fig. 2). The 5y-OS was 77.3% (95% CI 49.0–91.1%) for those without extramedullary disease (n = 21) and was 80.8% (95% CI 42.3–94.9%) for those with extramedullary disease (n = 11), and these were not significantly different (*p* = 0.561).

**Table 3:** Patient characteristics and outcomes for children with de novo BP

Pt No.	Age/sex	Phenotype	Extramedullary disease	Pre-HSCT TKIs	Pre-HSCT treatment	Pre-HSCT state	Conditioning	Graft	Donor	Post-HSCT TKIs	Current treatment	Final CML state	Outcome
BP-1	10/F	L	Subcutaneous + paravertebral	No	HU, IFNα	BP/NHR	MAC	BMT	MSD	No	(Death on disease)	BP	Dead
BP-2	8/F	NA	Subcutaneous	No	HU, IFNα, BU	AP	MAC	BMT	MUD	No	(NRM due to aGVHD, renal failure, ICH)	CHR	Dead
BP-3	14/M	NA	Intramuscular	No	HU	PHR	MAC	BMT	MSD	No	TKI–	CCyR	Alive
BP-4	5/F	M	No	No	IFNα, AML-type chemo	PHR	MAC	BMT	MMUD	No	(NRM due to interstitial pneumonia)	CMR	Dead
BP-5	4/F	M	No	No	AraC	CHR	MAC	CBT	MMUD	No	(Death due to 2nd malig.)	CMR	Dead
BP-6	6/F	M	No	No	HU, IFNα, AraC	CCyR	MAC	BMT	MSD	No	TKI–	CMR	Alive
BP-7	8/M	NA	Orbital + subcutaneous	No	HU, IFNα	CCyR	MAC	BMT	MUD	ima^a^	TKI +	CMR	Alive
BP-8	10/M	L	No	Yes	Imatinib + ALL-type chemo	CCyR	NA	NA	NA	NA	NA	MMR	Alive
BP-9	2/F	L	No	Yes	Imatinib + ALL-type chemo	BP	MAC	BMT	MMRD	No	(NRM due to respiratory failure)	CMR	Dead
BP-10	13/M	L	No	Yes	Imatinib + ALL-type chemo	CHR	MAC	CBT	MMUD	ima → dasa^a^	2nd HSCT, TKI- (low dose Ara-C)	BP	Alive
BP-11	14/M	NA	Subcutaneous + hepatomegaly	Yes	Imatinib + AraC	PCyR	RIC	BMT	MUD	No	TKI-	CMR	Alive
BP-12	11/F	L	No	Yes	Imatinib + ALL-type chemo	PCyR	MAC	PBSCT	MMRD	No	TKI-	CMR	Alive
BP-13	2/M	L	No	Yes	Imatinib, Dasatinib + ALL-type chemo	CCyR	MAC	BMT	MMRD	dasa^b^	TKI-	MR4.5	Alive
BP-14	13/M	M	No	Yes	Imatinib + ALL-type chemo	CCyR	MAC	BMT	MMRD	No	TKI-	CCyR	Alive
BP-15	4/M	L	Subcutaneous	Yes	Imatinib	CCyR	RIC	BMT	MSD	No	TKI-	MR4.5	Alive
BP-16	13/F	M	Subcutaneous	Yes	Imatinib + HU	CCyR	MAC	PBSCT	MSD	No	TKI-	CCyR	alive
BP-17	12/F	M	Cranial bone	Yes	Imatinib	CCyR	MAC	PBSCT	MRD	No	TKI-	MR4.5	Alive
BP-18	13/M	NA	Testicular	Yes	Imatinib + Irradiation	CCyR	MAC	CBT	MUD	ima^b^ → dasa^a^	TKI- after 2nd HSCT	MMR	Alive
BP-19	4/M	L	No	Yes	Imatinib + ALL-type chemo	MMR	MAC	BMT	MSD	ima^b^	TKI-	MR4.5	Alive
BP-20	12/F	M	No	Yes	Imatinib	MMR	MAC	PBSCT	MRD	dasa^b^	TKI-	MR4.0	Alive
BP-21	8/F	M	No	Yes	Imatinib + AML-type chemo	MMR	RIC	CBT	MMUD	No	TKI-	MMR	Alive
BP-22	9/F	L	No	Yes	Imatinib + chemo (AML > ALL type)	MR4.0	MAC	CBT	MMUD	ima^b^	TKI-	MR4.5	Alive
BP-23	3/F	L	No	Yes	Imatinib + ALL-type chemo	MR4.5	RIC	BMT	MUD	no	TKI-	MR4.5	Alive
BP-24	10/F	L	No	Yes	Imatinib, Dasatinib + chemo	–	–	–	–	–	Preparing HSCT	Unknown	Alive
BP-25	10/F	L	No	Yes	Imatinib, Dasatinib + ALL-type chemo	CHR	MAC	CBT	MUD	dasa^b^	TKI-	MR4.0	Alive
BP-26	7/M	L	No	Yes	Imatinib, Dasatinib + ALL-type chemo	MR4.0	MAC	BMT	MMRD	dasa^a^	(NRM due to lung GVHD)	MR4.0	Dead
BP-27	6/M	M	[ +], site not available	Yes	Imatinib, Dasatinib, Nilotinib + HU, VP	CCyR	MAC	BMT	MMUD	No	TKI-	MR4.5	Alive
BP-28	16/M	M	[ +], site not available	Yes	Dasatinib + AML-type chemo	CCyR	MAC	BMT	MSD	dasa^b^	TKI + (dasa; prophylactic)	Unknown	Alive
BP-29	1/M	L	No	Yes	Dasatinib + ALL-type chemo	CCyR	MAC	BMT	MUD	dasa^a^ → ima^a^	TKI + (nilo; therapeutic)	MR4.0	Alive
BP-30	9/M	L	No	Yes	Dasatinib + ALL-type chemo	MMR	MAC	BMT	MSD	dasa^b^	TKI + (dasa; prophylactic)	MR4.5	Alive
BP-31	10/M	Mixed	No	Yes	Dasatinib + ALL-type chemo	MMR	RIC	BMT	MMRD	ima^b^	(NRM due to EBV-LPD)	MMR	Dead
BP-32	6/F	L	No	Yes	Dasatinib + ALL-type chemo	MR4.0	MAC	BMT	MSD	dasa^a^	TKI–	MR4.5	Alive

*BP* blast phase, *HSCT* hematopoietic stem cell transplantation, *TKI* tyrosine kinase inhibitor, *CML* chronic myeloid leukemia, *M* male, *F* female, *L* lymphoid, *M* myeloid, *HU* hydroxyurea, *IFNα* interferon α, *BU* busulfan, *AML* acute myeloid leukemia, *ALL* acute lymphoblastic leukemia, *NHR* no hematologic response, *AP* accelerated phase, *PHR* partial hematologic response, *CHR* complete hematologic response, *CCyR* complete cytogenetic response, *PCyR* partial cytogenetic response, *MMR* major molecular response, *MAC* myeloablative conditioning, *RIC* reduced intensity conditioning, *BMT* bone marrow transplantation, *CBT* cord blood transplantation, *PBSCT* peripheral blood stem cell transplantation, *MSD* matched sibling donor, *MMRD* mismatched related donor, *MUD* matched unrelated donor; ima, imatinib; dasa, dasatinib; nilo, nilotinib, *NRM* non-relapse mortality, *aGVHD* acute graft-versus-host disease, *ICH* intracranial hemorrhage, *EBV-LPD* EB virus-associated lymphoproliferative disease, *NA* not available

^a^TKIs were initiated after confirming disease recurrence (therapeutic)

^b^these TKIs were started before the recurrence of the disease (= prophylactic)

The initial treatments administered to the seven patients diagnosed during the pre-TKI era were heterogeneous (Table 3). Hydroxyurea was the most frequently used (5/7, 71.4%), mainly in combination with interferon-α. Three patients received cytarabine or AML-type chemotherapy. All these patients underwent myeloablative conditioning within a median of 202 days after diagnosis (range 63–371 days). The disease status at HSCT was BP in one patient; AP, 1; PHR, 2; CHR, 1; and CCyR, 2. One patient (BP-1) who received HSCT in BP died of CML, and three patients died of adverse events after undergoing transplantation (BP-4, interstitial pneumonia; BP-5, second malignancy [rhabdomyosarcoma]; BP-2, acute graft-versus-host disease [aGVHD]). Among three survivors, two patients achieved CMR with or without TKIs, and the remainder achieved CCyR without TKIs after HSCT at the last follow-up.

For all 25 patients diagnosed in the TKI era, TKIs were included in the initial therapy (imatinib, n = 20 and dasatinib, n = 5). In total, 21 patients received a combination of chemotherapy and TKIs, one patient received imatinib and irradiation, and three patients received imatinib as monotherapy. The details of chemotherapies are summarized in Table 3. The choice of chemotherapeutic agents was based on the immunophenotype of the disease: ALL-type chemotherapy was selected for those with a lymphoid phenotype, and AML-type chemotherapy or cytarabine was selected for those with a myeloid phenotype. Notably, one patient with lymphoid BP (BP-22) initially received AML-type chemotherapy; however, her therapy was changed to ALL-type chemotherapy because of a lack of response. She eventually achieved MR4.0 before receiving cord blood transplantation (CBT). Another patient with myeloid BP (BP-14) received ALL-type chemotherapy and achieved CCyR before receiving BMT.

Of the 25 patients diagnosed in the TKI era, 24 underwent HSCT, and the remaining patients (BP-24) planned to receive HSCT at the last follow-up of the study period. Among the 24 patients who had already received HSCT, the disease status of 19 treated with imatinib at transplantation was BP in 1; CHR, 2; PCyR, 2; CCyR, 8; MMR, 3; MR4.0, 2; and MR4.5, 1. Five patients who received dasatinib as first-line therapy achieved CCyR (n = 2), MMR (n = 2), and MR4.0 (n = 1) before HSCT. HSCT (BMT, n = 14; PBSCT, n = 4; CBT, n = 5) was performed using MSD (n = 6), HLA-matched related donors (MRD) (n = 2), MMRD (n = 6), MUD (n = 5), and MMUD (n = 4), at a median of 221 days after diagnosis (range 112–373 days). Transplantation conditioning regimens were myeloablative in 18 patients and reduced intensity in five patients. Information on neither the donor source nor the conditioning regimen was available for one patient. Three patients died of adverse events (BP-9, respiratory failure; BP-31, Epstein–Barr virus-associated lymphoproliferative disorder; BP-26, lung GVHD). Among the 20 patients who received HSCT and survived at the time of analysis, TKI administration could be stopped in nine patients after transplantation.

## Discussion

We analyzed the characteristics and outcomes of children with de novo AP and BP-CML in Japan. Although de novo advanced-phase CML is an extremely rare disease entity during childhood, the current study included a large number of patients comparable to those in previous large studies [3, 6]. Therefore, the current study adds clinically useful information regarding patient characteristics and the selection of treatment strategies.

In the current study, cohort A included children in all disease phases of CML between 1996 and 2011 in Japan; AP comprised 4.3% and BP comprised 6.6% of children with newly diagnosed CML during this period. The proportions of advanced-phase CML were slightly higher in our study than those in the I-CML-Ped cohort, in which AP was reported in 4% and BP in 3.5% of the included patients [6].

The background characteristics of the patients with AP-CML and BP-CML differed in some ways, but both showed excellent outcomes (Table 1 and Fig. 1). Male predominance was clearer in AP than that in BP, and this showed a different trend from the pediatric report from I-CML-Ped, where the male/female ratio was 12/7 in AP and 11/6 in BP and from the adult report from the Swedish registry, where the male/female ratio was 11/9 in AP and 13/3 in BP [6, 15]. Furthermore, several additional characteristics differed slightly from those reported in the European I-CML-Ped study as our cohort included younger patients with milder hematologic abnormalities [6]. Meanwhile, both our cohort and the I-CML-Ped cohort showed excellent patient outcomes; the 5-year OS for children with de novo AP was 93.3% in our cohort, whereas it was 94% in the I-CML-Ped cohort, and the 5-year OS for children with de novo BP was 79.0% in our cohort, whereas it was 74% in the I-CML-Ped cohort. Moreover, as expected, the outcomes of patients with AP- and BP-CML who received pre-HSCT TKIs were better than those of patients who did not receive pre-HSCT TKIs (Fig. 2).

One striking difference between our report and that of I-CML-Ped is the surprisingly high frequency of extramedullary BP-CML without bone marrow involvement in our cohort. The sites of extramedullary involvement are shown in Table 3, with subcutaneous masses being the most frequent (6/9; 66.7%). Although we could not identify the reason for the higher frequency of extramedullary involvement in the current study, these patients received conventional therapy and had favorable outcomes during the TKI era (Table 3).

The outcome of AP-CML was excellent, with a 100% 5-year OS for those receiving TKIs and subsequent HSCT, which was in line with the report from I-CML-Ped. In contrast, 11/15 patients (73.3%) received HSCT in our study, whereas only 6/19 (31.6%) patients received HSCT in the I-CML-Ped cohort. All four patients who did not receive HSCT in our cohort survived with a deep molecular response to TKI administration, with the median follow-up of 5.5 years (range 1.70–10.74, Table 2). One notable finding here is that two patients receiving 2G-TKIs (AP-14 and AP-15, Table 2) had been followed without HSCT at the time of the last follow-up. Moreover, one patient in our cohort died of transplantation-related thrombotic microangiopathy. These findings support the current recommendation that patients with de novo AP should be treated with TKIs and could avoid HSCT, particularly in cases with good treatment response [6, 16], and 2G-TKI would be preferable in this strategy, although this has not been systematically validated [16].

The treatment strategies for BP-CML were generally in line with the report from the I-CML-Ped, as approximately half of the patients received a combination of chemotherapy with TKIs in both cohorts. In the current cohort, most patients received chemotherapy according to their immunophenotype at diagnosis (i.e. ALL-type chemotherapy with TKI for children with lymphoid BP and AML-type chemotherapy with TKI for children with myeloid BP). Notably, one patient (BP-22) who initially received AML-type chemotherapy changed her therapy to ALL-type chemotherapy because of a lack of response and subsequently achieved MR4.0 before receiving HSCT. As such, treatment selection between ALL- or AML-type chemotherapy based on the immunophenotype at diagnosis seemed reasonable, as guided by the recent expert consensus [17]. A recent study from the Japanese Transplantation Registry identified that disease status at the molecular level at HSCT was strongly associated with patient outcomes in children with de novo BP-CML [18]; further optimization of treatment plans before HSCT is warranted.

Among children with advanced phase CML, who can avoid HSCT [6], and what is the optimal approach to incorporate post-HSCT TKIs, are two important questions. In the recent expert recommendation, HSCT with close molecular monitoring, such as monthly monitoring in the first year, was recommended for all children with BP-CML [17]. In the current study, the choice of post-HSCT TKI usage and the timing of discontinuation of post-transplant TKIs was completely based on treating physicians’ preferences, and we did not collect the information on the duration of TKIs. Although clinical characteristics including older age, low hemoglobin and platelet counts, high lactate dehydrogenase, myeloid immunophenotype were associated with inferior outcomes among adults with BP-CML [19, 20], risk factors for children with advanced phase CML has not been reported. Furthermore, a novel drug asciminib has shown the promising treatment response for adults with CML [21], and will potentially enable more patients with advanced phase CML to avoid HSCT. As the number of children with advanced phase CML is quite limited, international collaboration would be warranted to identify the factors which can guide avoidance of HSCT and can support the choice of post-HSCT TKIs.

One attractive option suggested by the current analysis is RIC for children with de novo BP-CML. In the current analysis for children with BP-CML receiving TKIs as first-line therapy (n = 25), five received the RIC regimen as a conditioning regimen, which resulted in four patients being alive. This result suggested that at least a part of children with de novo BP-CML can avoid MAC-HSCT and be cured with RIC-HSCT. Although the recent international study from I-CML-Ped suggested the potential avoidance of HSCT for children with BP-CML [6], at the same time in the expert consensus report, MAC-HSCT was recommended for all children with BP-CML [17]. Accordingly, RIC-HSCT can be an attractive option between no-HSCT and MAC-HSCT, but further data accumulation is warranted to clarify how to incorporate RIC into the strategy for children with de novo BP-CML.

The current study had several limitations. First, as this was a retrospective study, the treatment strategies applied were inconsistent among patients, making it difficult to show whether any of the treatment strategies were superior to the others. Moreover, the results might be biased by the retrospective nature of this study. Second, information regarding transcripts of *BCR::ABL1* or additional gene mutations that have been reported to influence patient outcomes is lacking [22, 23]. Third, CML diagnosis was based on the institutional diagnosis, and the diagnosis was not centrally reviewed, which could lead to unclear discrimination between BP-CML and acute leukemia with *BCR::ABL1*. Finally, the number of patients included in the study was small. However, as de novo AP-CML and BP-CML are extremely rare, we believe that this study provides clinically useful information.

In summary, this retrospective study confirmed excellent outcomes in children with de novo advanced-phase CML, and added clinically useful information regarding patient characteristics and the selection of treatment options. International collaboration is required to further optimize treatment strategies, particularly for children with de novo BP-CML.

## Data Availability

The data that support the findings of this study are available from the corresponding author upon reasonable request.
